# Pregnancy outcomes of dichorionic triamniotic triplet pregnancies after in vitro fertilization-embryo transfer: multifoetal pregnancy reduction versus expectant management

**DOI:** 10.1186/s12884-020-2815-4

**Published:** 2020-03-17

**Authors:** Pei Cai, Yan Ouyang, Fei Gong, Xihong Li

**Affiliations:** 1 Institute of Reproductive and Stem Cell Engineering, Central South University, Changsha City, 410078 Hunan China; 2 Reproductive and Genetic Hospital of CITIC-Xiangya, Changsha City, 410078 Hunan China

**Keywords:** Dichorionic triamniotic triplet pregnancies, Multifoetal pregnancy reduction, In vitro fertilization-embryo transfer, Pregnancy outcomes

## Abstract

**Background:**

Trichorionic triplet pregnancy reduction to twin pregnancy is associated with a lower risk of preterm delivery but not with a lower risk of miscarriage. However, data on dichorionic triamniotic (DCTA) triplet pregnancy outcomes are lacking. This study aimed to compare the pregnancy outcomes of DCTA triplets conceived via in vitro fertilization-embryo transfer (IVF-ET) managed expectantly or reduced to a monochorionic (MC) singleton or monochorionic diamniotic (MCDA) twins at 11–13^+ 6^ gestational weeks.

**Methods:**

Two hundred ninety-eight patients with DCTA triplets conceived via IVF-ET between 2012 and 2016 were retrospectively analysed. DCTA triplets with three live foetuses were reduced to a MC singleton (group A) or MCDA twins (group B) or underwent expectant management (group C). Each multifoetal pregnancy reduction (MFPR) was performed at 11–13^+ 6^ gestational weeks. Pregnancy outcomes in the 3 groups were compared.

**Results:**

Eighty-four DCTA pregnancies were reduced to MC singleton pregnancies, 149 were reduced to MCDA pregnancies, and 65 were managed expectantly.

There were no significant differences among groups A, B, and C in miscarriage rate (8.3 vs. 7.4 vs. 10.8%, respectively) and live birth rate (90.5 vs. 85.2 vs. 83.1%, respectively) (*P* > 0.05).

Group A had significantly lower rates of preterm birth (8.3 vs. 84.6%; odds ratio (OR) 0.017, 95% confidence interval (CI) 0.006–0.046) and low birth weight (LBW; 9.2 vs. 93.2%; OR 0.007, 95% CI 0.003–0.020) than group C (*P* < 0.001).

Group B had significantly lower preterm birth (47.0 vs. 84.6%; OR 0.161, 95% CI 0.076–0.340) and LBW rates (58.7 vs. 93.2%; OR 0.103, 95% CI 0.053–0.200) than group C (*P* < 0.001).

Group A had significantly lower preterm birth (8.3 vs. 47.0%; OR 0.103, 95% CI 0.044–0.237; *P* < 0.001), LBW (9.2 vs. 58.7%; OR 0.071, 95% CI 0.032–0.162; *P* < 0.001) and perinatal death rates (1.3 vs. 9.1%; OR 0.132, 95% CI 0.018–0.991; *P* = 0.021) than group B.

**Conclusion:**

The MFPR of DCTA triplets to singleton or MCDA pregnancies was associated with better pregnancy outcomes compared to expectant management. DCTA triplets reduced to singleton pregnancies had better perinatal outcomes than DCTA triplets reduced to MCDA pregnancies.

## Background

Over the past few decades, there has been a dramatic increase in the incidence of multifoetal pregnancies (MFPs) due to advancing maternal age, the widespread application of assisted reproductive technology (ART) and the use of ovulation induction drugs [1–3]. As a result of restrictions on the number of embryos transferred in women undergoing ART, a decline in MFPs has been observed in recent years [4, 5]. However, the splitting of one embryo into two embryos may lead to higher-order multiple pregnancies (HOMPs), including triplet pregnancies containing monochorionic (MC) twins [5–8].

Compared with singleton and twin pregnancies, HOMPs are associated with a higher risk of maternal-perinatal and long-term complications [9–12] and increased hospital costs [13]. Compared with singleton and twin pregnancies, triplet pregnancies are at a higher risk of miscarriage and preterm birth [5, 14–17]. To reduce the risks associated with triplet pregnancies and HOMPs [18, 19], multifoetal pregnancy reduction (MFPR) has been performed in recent years, and several methods have been described [20, 21]. There is ample evidence that reducing quadruplet-or-higher pregnancies to twins is associated with more favourable outcomes, including advanced gestational age (GA) at delivery [15, 16, 22]. A meta-analysis [23] showed that trichorionic triplet pregnancy reduction to a twin pregnancy is associated with a lower risk of preterm delivery with no significant increase in the miscarriage rate. However, data on the perinatal outcomes of women with dichorionic triamniotic (DCTA) triplet pregnancies who undergo MFPR are lacking, and two meta-analyses on this subject reported that the numbers are insufficient to recommend one technique over another or to draw clear conclusions on the perinatal outcomes of DCTA pregnancies [3, 23].

The aim of this study was to investigate the pregnancy and obstetric outcomes of women with DCTA pregnancies conceived by IVF-ET that were managed expectantly or were reduced to singleton pregnancies (foetus with a separate placenta) or monochorionic diamniotic (MCDA) twin pregnancies at 11–13^+ 6^ gestational weeks.

## Material and methods

### Patients

A retrospective analysis was conducted of infertile patients with DCTA pregnancies conceived via IVF-ET from January 2012 to December 2016 at the Reproductive and Genetic Hospital of CITIC-Xiangya (Changsha City, Hunan, China). This study was approved by the Ethics Committee of the Reproductive and Genetic Hospital of CITIC-Xiangya.

**Fig. 1:**
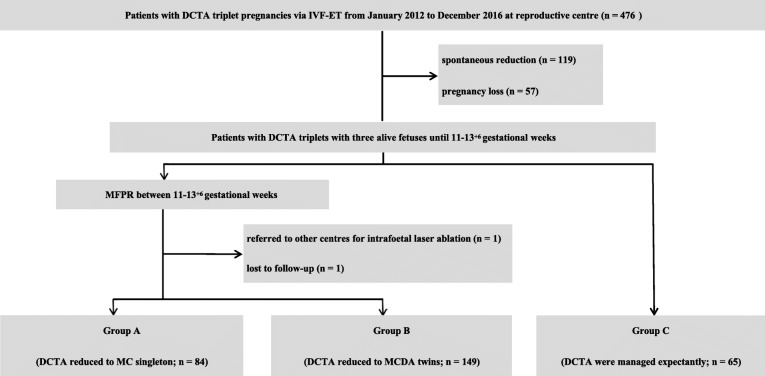
Flow diagram showing the cases included in among group A, B and C. *DCTA* dichorionic triamniotic, *IVT-ET* in vitro fertilization-embryo transfer, *MFPR* multifoetal pregnancy reduction, *MC* monochorionic, *MCDA* monochorionic diamniotic

We identified 476 infertile patients who conceived DCTA triplets via IVF-ET. The IVF and ET procedures were carried out as previously described [24]. Chorionicity was determined during the first trimester by ultrasound based on the number of placental sites, the presence of the “lambda sign” or “T sign” in a single placenta, and an evaluation of interfoetal membranes by experienced radiologists [25]. Only those who underwent IVF-ET, ultrasound examinations and MFPR at our hospital were included in this study. Patients who experienced spontaneous reductions or pregnancy loss (*n* = 176) before 11–13^+ 6^ gestational weeks, referred to other centers for intrafoetal laser ablation (*n* = 1) or were lost to follow-up (*n* = 1) were excluded from this analysis. Finally, 298 patients with DCTA pregnancies with three viable foetuses until 11–13^+ 6^ gestational weeks were included in the data analysis (Fig. 1). When a foetal heartbeat was detected by ultrasound, the pregnancy was considered viable. GA was based on the date of embryo transfer (ET) plus 17 or 19 days for day 3 embryo or blastocyst transfers, respectively.

### MFPR procedure

All patients were counselled regarding the risks of a DCTA pregnancy and the different management options, including MFPR (including the injection and vascular-occlusive techniques, such as intrafoetal laser ablation) and expectant management. Patients were informed in detail about the risks and benefits of MFPR. Given the lack of clear evidence regarding the best method for MFPR and the psychological impact of reduction on patients, the final choice was based mostly on patient preference. Because intrafoetal laser ablation was not performed at our centre, patients considered this technique were referred to a second centre. Patients who decided to undergo foetal reduction at our centre followed our routine foetal reduction procedure. The reasons for MFPR were either structural abnormalities (such as abnormal foetal nuchal translucency (NT), severe foetal cardiac malformations and foetal limb defects) in one or two of the foetuses or patient preference. Written informed consent for foetal reduction was obtained from each participant. The procedure was performed transabdominally by the ultrasound-guided intrathoracic injection of potassium chloride (10% KCl, 2 ml) using a 20 G spinal needle (15 cm in length). All reductions were performed by a highly skilled physician (Dr Yan Shen). MFPRs were performed at 11–13^+ 6^ gestational weeks (58–80 days after ET) after a NT scan. The selection of foetuses to be reduced was based on the NT scan and accessibility. If one or both of the MCDA twins in the DCTA pregnancy had an abnormal NT or (and) certain other structural abnormalities, both were reduced, and if the MC singleton of the DCTA pregnancy had an abnormal NT or (and) certain other structural abnormalities, this foetus was reduced. If the ultrasound scan showed no abnormality in any foetus, patient preference and foetal accessibility dictated the reduction of the MC singleton or MCDA twins. An abnormal (increased) NT was defined as a foetal NT of 3 mm or more detected by ultrasound examination in the first trimester [26].

### Outcome measures

Maternal demographics, ultrasound findings and IVF-ET and MFPR procedure details were recorded in the medical records. The pregnancy and obstetric outcomes were followed up by telephone or fax. The pregnancy and perinatal outcomes were defined as follows: miscarriage: pregnancy loss before 24 gestational weeks; preterm birth: delivery at a minimum of 24 gestational weeks but before 37 gestational weeks; very preterm birth (VPB): delivery at or later than 24 gestational weeks but before 32 gestational weeks; and term birth: delivery at or later than 37 gestational weeks but before 42 gestational weeks [5]. Additionally, intrauterine death (IUD) was defined as foetal demise from 24 gestational weeks. Perinatal death included IUD and neonatal death (NND) of live-born infant during the first 28 days [27]. Low birth weight (LBW) was defined as a birth weight of less than 2500 g, and very low birth weight (VLBW) was defined as a birth weight of less than 1500 g [28].

### Statistical analysis

Statistical analyses were conducted using SPSS version 17.0 software (SPSS, Inc., Chicago, IL, USA). Descriptive statistics are presented as the means ± standard deviations (SDs) and as percentages for enumerated data. Differences in the means between the two groups were analysed using Student’s t-test. The chi-squared test or Fisher’s exact test was used to determine the statistical significance of differences between percentages. Statistical significance was set at *P* < 0.05.

## Results

Eighty-four DCTA triplets were reduced to MC singleton pregnancies (group A), 149 DCTA triplet pregnancies were reduced to MCDA twin pregnancies (group B), and 65 DCTA pregnancies were managed expectantly (group C).

Groups A, B and C were statistically similar regarding maternal age (29.6 ± 4.2 vs. 29.4 ± 3.9 vs. 28.4 ± 3.7 years), body mass index (21.5 ± 2.7 vs. 21.7 ± 3.0 vs. 21.9 ± 3.1 kg/m^2^), infertility duration (4.4 ± 3.5 vs. 4.3 ± 2.8 vs. 4.1 ± 3.2 years), transfer cycles (1.2 ± 0.5 vs. 1.1 ± 0.5 vs. 1.1 ± 0.5), infertility type, cause of infertility, and insemination methods (*P* > 0.05) (Table 1).

**Table 1:** Comparison of the maternal demographic characteristics among groups A, B and C

characteristic	Group A	Group B	Group C	Group A vs. Group C	Group B vs. Group C	Group A vs. Group B
	(*n* = 84)	(*n* = 149)	(*n* = 65)	*P* - value	*P* - value	*P* - value
Maternal age (years)	29.6 ± 4.2	29.4 ± 3.9	28.4 ± 3.7	NS	NS	NS
BMI (kg/m^2^)	21.5 ± 2.7	21.7 ± 3.0	21.9 ± 3.1	NS	NS	NS
Infertility duration (years)	4.4 ± 3.5	4.3 ± 2.8	4.1 ± 3.2	NS	NS	NS
Infertility type				NS	NS	NS
Primary, n (%)	39 (46.4)	62 (41.6)	36 (55.4)			
Secondary, n (%)	45 (53.6)	87 (58.4)	29 (44.6)			
Cause of infertility				NS	NS	NS
Male factor , n (%)	58 (69.0)	94 (63.1)	47 (72.3)			
Female factor , n (%)	3 (3.6)	3 (2.0)	1 (1.5)			
Female + male factors, n (%)	23 (27.4)	49 (32.9)	14 (21.5)			
Unexplained, n (%)	0 (0)	3 (2.0)	3 (4.6)			
Transfer cycle	1.2 ± 0.5	1.1 ± 0.5	1.1 ± 0.5	NS	NS	NS
1, n (%)	72 (85.7)	135 (90.6)	58 (89.2)	NS	NS	NS
≥2, n (%)	12 (14.3)	14 (9.4)	7 (10.8)			
Insemination methods				NS	NS	NS
IVF, n (%)	49 (58.3)	96 (64.4)	45 (69.2)			
ICSI, n (%)	9 (10.7)	20 (13.4)	9 (13.8)			
IVF/ICSI, n (%)	26 (31.0)	33 (22.1)	11 (16.1)			

Group A = DCTA pregnancy reduced to MC singleton pregnancy; Group B = DCTA pregnancy reduced to MCDA twin pregnancy; Group C = DCTA pregnancy were managed expectantly

*DCTA* dichorionic triamniotic, *MC* monochorionic, *MCDA* monochorionic diamniotic, *BMI* body mass index, *NS* not significant, *IVF* in vitro fertilization, *ICSI* intracytoplasmic sperm injection

Group A had significantly lower rates of preterm birth (8.3 vs. 84.6%; odds ratio (OR) 0.017, 95% confidence interval (CI) 0.006–0.046), VPB (2.6 vs. 22.4%; OR 0.092, 95% CI 0.020–0.428), LBW (9.2 vs. 93.2%; OR 0.007, 95% CI 0.003–0.020) and perinatal death (1.3 vs. 9.8%; OR 0.122, 95% CI 0.016–0.930) than group C (*P* < 0.05). GA at delivery (38.5 ± 2.1 vs. 33.4 ± 3.0 weeks) and live birth weight (3168 ± 557 vs. 1827 ± 441 g) were significantly higher in group A than in group C (*P* < 0.001). There was no significant difference in the miscarriage rate (8.3 vs. 10.8%) and the live birth rate (90.5 vs. 83.1%) between groups A and C (*P* > 0.05) (Table 2).

**Table 2:** Pregnancy and obstetric outcomes in group A and group C

Pregnancy outcomes	Group A	Group C	*P*-value	OR (95% CI)
Pregnancy	84	65		
Miscarriage rate, % (n)	8.3 (7/84)	10.8 (7/65)	0.613	0.753 (0.250–2.267)
Preterm birth rate, % (n)	8.3 (7/84)	84.6 (55/65)	< 0.001	0.017 (0.006–0.046)
Term birth rate, % (n)	83.3 (70/84)	4.6 (3/65)	< 0.001	103.333 (28.361–376.496)
Caesarean section rate, % (n)	68.8 (53/77)	87.9 (51/58)	0.009	0.303 (0.120–0.765)
Babies born	77	174		
Live births	76	162		
Live birth rate, % (n)	90.5 (76/84)	83.1 (162/195)	0.109	1.935 (0.853–4.390)
Perinatal death, % (n)	1.3 (1/77)	9.8 (17/174)	0.016	0.122 (0.016–0.930)
IUD rate, % (n)	1.3 (1/77)	6.9 (12/174)	0.071	0.178 (0.023–1.391)
NND rate, % (n)	0 (0/77)	2.9 (5/174)	0.327	–
Gestational age at delivery (weeks)	38.5 ± 2.1	33.4 ± 3.0	< 0.001	
≥ 37 weeks, % (n)	90.9 (70/77)	5.2 (3/58)	< 0.001	183.333 (45.307–741.858)
< 37 weeks, % (n)	9.1 (7/77)	94.8 (55/58)	< 0.001	0.005 (0.001–0.022)
VPB 24-31^+6^ weeks, % (n)	2.6 (2/77)	22.4 (13/58)	< 0.001	0.092 (0.020–0.428)
Live birth weight (g)	3168 ± 557	1827 ± 441	< 0.001	
≥ 2500 g, % (n)	90.8 (69/76)	6.8 (11/162)	< 0.001	135.312 (50.304–363.975)
LBW < 2500 g, % (n)	9.2 (7/76)	93.2 (151/162)	< 0.001	0.007 (0.003–0.020)
VLBW < 1500 g, % (n)	0 (0/76)	17.9 (29/162)	< 0.001	–

Group A = DCTA pregnancy reduced to MC singleton pregnancy; Group C = DCTA pregnancy were managed expectantly

*DCTA* dichorionic triamniotic, *MC* monochorionic, *OR* odds ratio, *CI* confidence interval, *IUD* intrauterine death, *NND* neonatal death, *VPB* very preterm birth, *LBW* low birth weight, *VLBW* very low birth weight

Group B had significantly lower rates of preterm birth (47.0 vs. 84.6%; OR 0.161, 95% CI 0.076–0.340), VPB (7.2 vs. 22.4%; OR 0.270, 95% CI 0.111–0.660) and LBW (58.7 vs. 93.2%; OR 0.103, 95% CI 0.053–0.200) than group C (*P* < 0.05). In addition, GA at delivery (35.7 ± 3.1 vs. 33.4 ± 3.0 weeks) and live birth weight (2348 ± 488 vs. 1827 ± 441 g) were significantly higher in group B than in group C (*P* < 0.001). There was no significant difference in the miscarriage rate (7.4 vs. 10.8%) and the live birth rate (85.2 vs. 83.1%) between groups B and C (*P* > 0.05) (Table 3).

**Table 3:** Pregnancy and obstetric outcomes in group B and group C

Pregnancy outcomes	Group B	Group C	*P*-value	OR (95% CI)
Pregnancy	149	65		
Miscarriage rate, % (n)	7.4 (11/149)	10.8 (7/65)	0.412	0.660 (0.244–1.788)
Preterm birth rate, % (n)	47.0 (70/149)	84.6 (55/65)	< 0.001	0.161 (0.076–0.340)
Term birth rate, % (n)	45.6 (68/149)	4.6 (3/65)	< 0.001	17.350 (5.212–57.756)
Caesarean section rate, % (n)	86.2 (119/138)	87.9 (51/58)	0.749	0.860 (0.340–2.171)
Babies born	276	174		
Live births	254	162		
Live birth rate, % (n)	85.2 (254/298)	83.1 (162/195)	0.519	1.176 (0.719–1.924)
Perinatal death, % (n)	9.1 (25/276)	9.8 (17/174)	0.8	0.920 (0.481–1.758)
IUD rate, % (n)	8.0 (22/276)	6.9 (12/174)	0.674	1.169 (0.563–2.427)
NND rate, % (n)	1.1 (3/276)	2.9 (5/174)	0.270	0.371 (0.088–1.574)
Gestational age at delivery (weeks)	35.7 ± 3.1	33.4 ± 3.0	< 0.001	
≥ 37 weeks, % (n)	49.3 (68/138)	5.2 (3/58)	< 0.001	17.810 (5.316–59.665)
< 37 weeks, % (n)	50.7 (70/138)	94.8 (55/58)	< 0.001	0.056 (0.017–0.188)
VPB 24-31^+6^ weeks, % (n)	7.2 (10/138)	22.4 (13/58)	0.003	0.270 (0.111–0.660)
Live birth weight (g)	2348 ± 488	1827 ± 441	< 0.001	
≥ 2500 g, % (n)	41.3 (105/254)	6.8 (11/162)	< 0.001	9.674 (4.994–18.737)
LBW < 2500 g, % (n)	58.7 (149/254)	93.2 (151/162)	< 0.001	0.103 (0.053–0.200)
VLBW < 1500 g, % (n)	4.3 (11/254)	17.9 (29/162)	< 0.001	0.208 (0.100–0.429)

Group B = DCTA pregnancy reduced to MCDA twin pregnancy; Group C = DCTA pregnancy were managed expectantly

*DCTA* dichorionic triamniotic, *MCDA* monochorionic diamniotic, *OR* odds ratio, *CI* confidence interval, *IUD* intrauterine death, *NND* neonatal death, *VPB* very preterm birth, *LBW* low birth weight, *VLBW* very low birth weight

Group A had significantly lower rates of preterm birth (8.3 vs. 47.0%; OR 0.103, 95% CI 0.044–0.237), LBW (9.2 vs. 58.7%; OR 0.071, 95% CI 0.032–0.162) and perinatal death (1.3 vs. 9.1%; OR 0.132, 95% CI 0.018–0.991) than group B (*P* < 0.05). Additionally, GA at delivery (38.5 ± 2.1 vs. 35.7 ± 3.1 weeks) and live birth weight (3168 ± 557 vs. 2348 ± 488 g) were significantly higher in group A than in group B (*P* < 0.001). However, there was no significant difference in the miscarriage rate (8.3 vs. 7.4%) and the live birth rate (90.5 vs. 85.2%) between groups A and B (*P* > 0.05) (Table 4).

**Table 4:** Pregnancy and obstetric outcomes in group A and group B

Pregnancy outcomes	Group A	Group B	*P*-value	OR (95% CI)
Pregnancy	84	149		
Miscarriage rate, % (n)	8.3 (7/84)	7.4 (11/149)	0.794	1.140 (0.425-3.063)
Preterm birth rate, % (n)	8.3 (7/84)	47.0 (70/149)	<0.001	0.103 (0.044-0.237)
Term birth rate, % (n)	83.3 (70/84)	45.6 (68/149)	<0.001	5.956 (3.084-11.502)
Caesarean section rate, % (n)	68.8 (53/77)	86.2 (119/138)	0.002	0.353 (0.178-0.698)
Babies born	77	276		
Live births	76	254		
Live birth rate, % (n)	90.5 (76/84)	85.2 (254/298)	0.216	1.646 (0.743-3.647)
Perinatal death, % (n)	1.3 (1/77)	9.1 (25/276)	0.021	0.132 (0.018-0.991)
IUD rate, % (n)	1.3 (1/77)	8.0 (22/276)	0.036	0.152 (0.020-1.146)
NND rate, % (n)	0 (0/77)	1.1 (3/276)	1.000	-
Gestational age at delivery (weeks)	38.5 ± 2.1	35.7 ± 3.1	<0.001	
≥ 37 weeks, % (n)	90.9 (70/77)	49.3 (68/138)	<0.001	10.294 (4.419-23.979)
< 37 weeks, % (n)	9.1 (7/77)	50.7 (70/138)	<0.001	0.097 (0.042-0.226)
VPB 24-31^+6^ weeks, % (n)	2.6 (2/77)	7.2 (10/138)	0.219	0.341 (0.073-1.600)
Live birth weight (g)	3168 ± 557	2348 ± 488	<0.001	
≥ 2500 g, % (n)	90.8 (69/76)	41.3 (105/254)	<0.001	13.988 (6.182-31.651)
LBW < 2500 g, % (n)	9.2 (7/76)	58.7 (149/254)	<0.001	0.071 (0.032-0.162)
VLBW < 1500 g, % (n)	0 (0/76)	4.3 (11/254)	0.075	-

Group A = DCTA pregnancy reduced to MC singleton pregnancy; Group B = DCTA pregnancy reduced to MCDA twin pregnancy

*DCTA* dichorionic triamniotic, *MC* monochorionic, *MCDA* monochorionic diamniotic, *OR* odds ratio, *CI* confidence interval, *IUD* intrauterine death, *NND* neonatal death, *VPB* very preterm birth, *LBW* low birth weight, *VLBW* very low birth weight

## Discussion

In the present study, we analysed the pregnancy and obstetric outcomes of women with DCTA pregnancies conceived by IVF-ET who underwent MFPR at 11–13^+ 6^ gestational weeks or expectant management. We found that the MFPR of DCTA pregnancies to either MC singleton or MCDA twin pregnancies improved the pregnancy and obstetric outcomes by significantly decreasing the risks of preterm birth, VPB and LBW and significantly increasing the GA at delivery and live birth weight, with no significant reduction in the miscarriage risk. Specifically, among the management options, the reduction of DCTA pregnancies to MC singleton pregnancies resulted in the lowest risks for VPB, perinatal death and LBW and in maximal GA at delivery and live birth weight.

Women with DCTA pregnancies carry both the risks associated with triplets, such as VPB, selective growth restriction and foetal malformation, and those associated with MC twins due to vascular anastomoses in the single placental bed, such as twin-to-twin transfusion syndrome (TTTS) and selective intrauterine growth restriction (SIUGR) [5, 29]. Patients should be informed in detail about all possible complications. Data from previous studies [30, 31] demonstrated that MFPR is feasible and effective at decreasing the risk of some adverse outcomes for pregnancies with MC twins.

The most frequently applied method for MFPR is the ultrasound-guided transabdominal injection of KCl into the foetal heart or thoracic cavity, which has been shown to be relatively safe [32]. In the present study, MFPR was performed for 233 DCTA pregnancies using the injection technique. Ultrasound examination within 24 h of the procedure demonstrated that all retained MC singletons or MCDA twins were alive, and only 1.3% (3/233) of cases (2 cases of DCTA pregnancy reduced to a singleton pregnancy and 1 case of DCTA pregnancy reduced to a MCDA twin pregnancy) resulted in miscarriage in the subsequent 2 weeks. The procedure was technically successful in all cases.

Some studies comparing expectant management to the reduction of dichorionic (DC) triplet pregnancies to MC singleton pregnancies, foetal reduction resulted in a significantly decreased risk of preterm birth (< 32 gestational weeks), a more advanced GA at delivery and an increased birth weight, as well as a non-significantly increased risk of miscarriage (< 24 gestational weeks) [5, 30, 33]. Similarly, the present data showed that in DCTA pregnancies that were reduced to singleton pregnancies, the VPB rate decreased from 22.4 to 2.6%, the GA at delivery increased from 33.4 weeks to 38.5 weeks, and the live birth weight increased from 1827 g to 3168 g; the impact of foetal reduction on the miscarriage rate (8.3 vs. 10.8%) was limited.

A previous systematic review and meta-analysis compared the reduction of a DCTA pregnancy to a MC twin pregnancy (*n* = 15) with expectant management (*n* = 200) and found neither a significant increase in the risk of miscarriage (< 24 gestational weeks; 13.3 vs. 8.5%, respectively) nor a significant decrease in the risk of preterm birth (< 34 weeks; 46.2 vs. 51.9%, respectively) [23]. In contrast, the present study showed a significant decrease in the VPB rate from 22.4 to 7.2% and a slight decrease in the miscarriage rate from 10.8 to 7.4% among DCTA pregnancies reduced to MCDA pregnancies compared to expectant management. The differences in outcomes between the meta-analysis and the present study are potentially due to an inadequate number of patients with DCTA pregnancies reduced to MC twin pregnancies in the meta-analysis.

Reduction by the injection technique is not appropriate in women with a MC twin pregnancy because of inter-twin placental vascular anastomoses. In addition, acute haemorrhage of the surviving twin may occur soon after the death of the co-twin through placental vascular anastomoses, with consequent death or neurodevelopmental impairment [34, 35]. Women with DCTA triplet pregnancies reduced to MCDA pregnancies were exposed to the risks of TTTS and SIUGR. Relatively new vascular occlusive techniques have enabled the possibility of reducing a triplet pregnancy containing MC twins to a DC twin pregnancy [3, 36, 37]. Some studies have reported the efficiency of this new technique in women with MC twins; however, it potentially increases the risk of technique-associated complications and the rate of intrauterine demise of the retained co-twin [3, 34, 36]. Chaveeva P et al. [38] reported 61 DC triplet pregnancies that were reduced to DC twin pregnancies by intrafoetal laser ablation; although reduction resulted in a relatively lower miscarriage rate (3%), 45.9% of the cases of co-twin miscarriage within the subsequent 2 weeks were likely due to incomplete vascular occlusion and retrograde haemorrhage of the survivor through placental vascular anastomoses into the dead co-twin.

Rong Li et al. [31] reported that the MFPR of DC triplet pregnancies to singleton pregnancies had better pregnancy outcomes than those reduced to DC twin pregnancies by early transvaginal embryo reduction. The safety of a singleton pregnancy was also demonstrated in the present study. Our data showed that the reduction of DCTA pregnancies to singleton pregnancies decreased the risks of LBW and perinatal death and further increased the live birth weight compared with the reduction to MCDA pregnancies. However, two meta-analyses on the perinatal outcomes of management options for DCTA triplet pregnancies (including expectant management, reduction of the MC twins, reduction of one MC twin and reduction of the foetus with a separate placenta) reported that the number of cases was insufficient to recommend one technique or management method over another or to draw definitive conclusions on perinatal outcomes [3, 23]. For foetal reduction in DCTA triplet pregnancies, the current choice is mostly based on technical considerations and available instrumentation.

To our knowledge, this is the largest study to examine the outcomes of patients with DCTA pregnancies conceived via IVF-ET who underwent MFPR or expectant management. However, there are some limitations of this study. One limitation is the lack of data regarding morbidity among live infants, which is obviously more important than the live birth rate alone, and successful ART is defined as the delivery of a healthy and living baby by an infertile patient. In addition, our centre is only a reproductive centre, and all pregnancy outcomes were determined by telephone call or fax; therefore, we do not have reliable information about other pregnancy complications, such as gestational hypertension, gestational diabetes and premature rupture of membranes, or data on the frequency of TTTS in DCTA pregnancies reduced to MCDA pregnancies or managed expectantly. This was a retrospective analysis, and we probably missed some information regarding women who conceived DCTA triplets.

## Conclusion

In summary, in women with DCTA pregnancies conceived by IVF-ET who underwent MFPR at 11–13^+ 6^ gestational weeks or were managed expectantly, reduction to either singleton or MCDA pregnancies resulted in better pregnancy outcomes than expectant management. The perinatal outcomes of DCTA pregnancies reduced to singleton pregnancies were better than those of DCTA pregnancies reduced to MCDA pregnancies. Our data can assist physicians in counselling patients with DCTA pregnancies conceived by IVF-ET; however, reduction is a remedial tool to decrease the risks of a MFP. We recognize that the most effective measure to prevent unnecessary MFPs is to restrict the number of embryos transferred in women undergoing ART and to encourage selective single-blastocyst transfers.

## Data Availability

The data analysed during this study are included in the tables in this published article. The datasets used during the current study are available from the corresponding author on reasonable request.

## Abbreviations

ART: Assisted reproductive technology
CI: Confidence interval
DC: Dichorionic
DCTA: Dichorionic triamniotic
ET: Embryo transfer
GA: Gestational age
HOMPs: Higher-order multiple pregnancies
IUD: Intrauterine death
IVF-ET: In vitro fertilization-embryo transfer
LBW: Low birth weight
MC: Monochorionic
MCDA: Monochorionic diamniotic
MFP: Multifoetal pregnancy
NND: Neonatal death
NT: Nuchal translucency
OR: Odds ratio
SDs: Standard deviations
SIUGR: Selective intrauterine growth restriction
TTTS: Twin-to-twin transfusion syndrome
VLBW: Very low birth weight
VPB: Very preterm birth
